# Truncated O-Glycan-Bearing MUC16 Enhances Pancreatic Cancer Cells Aggressiveness via α4β1 Integrin Complexes and FAK Signaling

**DOI:** 10.3390/ijms23105459

**Published:** 2022-05-13

**Authors:** Christabelle Rajesh, Satish Sagar, Ashok Kumar Rathinavel, Divya Thomas Chemparathy, Xianlu Laura Peng, Jen Jen Yeh, Michael A. Hollingsworth, Prakash Radhakrishnan

**Affiliations:** 1 Eppley Institute for Research in Cancer and Allied Diseases, University of Nebraska Medical Center, Omaha, NE 68198-6805, USA; christabelle.rajesh@unmc.edu (C.R.); satish.sagar@unmc.edu (S.S.); a.rathinavel@unmc.edu (A.K.R.); divya.thomas@unmc.edu (D.T.C.); mahollin@unmc.edu (M.A.H.); 2 Lineberger Comprehensive Cancer Center, University of North Carolina at Chapel Hill, Chapel Hill, NC 27514-7295, USA; laura.peng@med.unc.edu (X.L.P.); jen_jen_yeh@med.unc.edu (J.J.Y.); 3 Fred & Pamela Buffett Cancer Center, University of Nebraska Medical Center, Omaha, NE 68198-6805, USA; 4 Department of Biochemistry and Molecular Biology, University of Nebraska Medical Center, Omaha, NE 68198-6805, USA

**Keywords:** MUC16, integrins, FAK, migration, pancreatic cancer

## Abstract

Elevated levels of Mucin-16 (MUC16) in conjunction with a high expression of truncated O-glycans is implicated in playing crucial roles in the malignancy of pancreatic ductal adenocarcinoma (PDAC). However, the mechanisms by which such aberrant glycoforms present on MUC16 itself promote an increased disease burden in PDAC are yet to be elucidated. This study demonstrates that the CRISPR/Cas9-mediated genetic deletion of MUC16 in PDAC cells decreases tumor cell migration. We found that MUC16 enhances tumor malignancy by activating the integrin-linked kinase and focal adhesion kinase (ILK/FAK)-signaling axis. These findings are especially noteworthy in truncated O-glycan (Tn and STn antigen)-expressing PDAC cells. Activation of these oncogenic-signaling pathways resulted in part from interactions between MUC16 and integrin complexes (α4β1), which showed a stronger association with aberrant glycoforms of MUC16. Using a monoclonal antibody to functionally hinder MUC16 significantly reduced the migratory cascades in our model. Together, these findings suggest that truncated O-glycan containing MUC16 exacerbates malignancy in PDAC by activating FAK signaling through specific interactions with α4 and β1 integrin complexes on cancer cell membranes. Targeting these aberrant glycoforms of MUC16 can aid in the development of a novel platform to study and treat metastatic pancreatic cancer.

## 1. Introduction

The tumor-associated antigen, CA125, is a routinely employed clinical biomarker for monitoring ovarian cancer progression [1,2]. CA125 is an epitope on Mucin-16 (MUC16), a massive (>22,000 amino acids) membrane-bound glycoprotein that is normally expressed in the epithelium of the endometrium, trachea, and cornea and is often upregulated in adenocarcinomas [3,4]. The full-length MUC16 protein contains a heavily glycosylated extracellular domain (ECD) with more than 60 tandem repeats (TR) that harbor binding sites for MUC16-specific antibodies and at least 56 repeated mucin-type domains that contain sea urchin, enterokinase, and agrin (SEA) repeats containing potential proteolytic cleavage sites [5]. Importantly, understanding the role of MUC16 in PDAC becomes pertinent because of its aberrant overexpression in more than 65% of PDAC cases [3]. Malignant transformation, a crucial hallmark of cancer, is often associated with aberrant glycosylation of proteins [6,7]. Mucin-type truncated O-glycosylation structures, such as the Tn (GalNAc-α-Ser/Thr) and STn (sialyl α2-6 GalNAc-α-Ser/Thr) antigens, are aggressively upregulated in most cancers [8,9]. The appearance of tumor-specific Tn and STn antigens occurs in more than 80% of human carcinomas, and in all cases, detection of the STn antigen correlates with poor prognosis and decreased overall survival [10,11]. Interestingly, overexpression of the STn antigen occurs at the highest frequency in pancreatic cancer [12]. Studies previously published by our group and other laboratories have demonstrated that induced expression of truncated Tn and STn antigens on mucins through knockout of the *COSMC* gene, a unique chaperone required for the function of the enzyme, C1GalT1, enhances the malignant potential of PDAC cells [13,14]. As MUC16 is one of the aberrantly O-glycosylated glycoproteins [15], we sought to investigate the involvement of such truncated O-glycans containing MUC16 in cellular mechanisms that can potentiate pancreatic tumorigenicity.

Cell migration is a dynamic multi-step process regulated by the balance between assembly and disassembly of cell adhesion molecules. Acquisition of migratory and invasive behavior is a necessary characteristic that promotes tumor progression and metastasis. Focal adhesion kinase (FAK) mediates a signaling cascade that has been implicated in a plethora of human cancers in the context of cell migration and invasion [16,17]. FAK is shown to directly bind to β-subunits of integrins involved in cell adhesion complexes and instigate a range of downstream signaling cascades, including PI3K/AKT [18,19]. In PDAC, overexpression of FAK and its increased phosphorylation at the active site, Tyrosine 397 (Y397), has been previously reported [20]. In the present study, we put forth a mechanism underlying the highly invasive behavior of PDAC cells expressing truncated O-glycan containing MUC16. Specifically, we demonstrated that the activation of the integrin-mediated focal adhesion kinase axis is upregulated in cases with such altered glycosylation of MUC16 to facilitate the cell migration and potential spread of PDAC.

## 2. Results

### 2.1. MUC16 Promotes PDAC Malignancy

The Clinical Proteomic Tumor Analysis Consortium (CPTAC) Discovery and Confirmatory dataset revealed strong upregulation of the MUC16 protein in high-grade PDAC tumors and the different stages of PDAC compared to the normal pancreas (Supplementary Figure S1A,B). There was a significant association between high MUC16 expression in a cohort of 116 PDAC patients and altered receptor-tyrosine kinase (RTK) pathways, a highly upregulated and tumor-promoting mechanism in this cancer (Supplementary Figure S1C) [21,22]. Further, in silico studies with The Cancer Genome Atlas (TCGA)-PAAD pancreatic ductal adenocarcinoma (PDAC) dataset [23] revealed a positive correlation between MUC16 and 636 other genes (threshold set to exclude genes with median transcripts per million (TPM) < 0.5) [24]. mRNA levels of crucial pro-metastatic genes in PDAC, like Annexin A1 (ANXA1) [25], showed a Pearson correlation coefficient of 0.54, indicating a strong probability for MUC16 dependent ANXA1 regulation to facilitate pro-tumoral function (Supplementary Table S1). Together, these results suggest that MUC16 expression is highly associated with malignant PDAC.

### 2.2. Truncated O-Glycan Containing MUC16 Promotes Migratory Behavior in PDAC Cells

**Figure 1:**
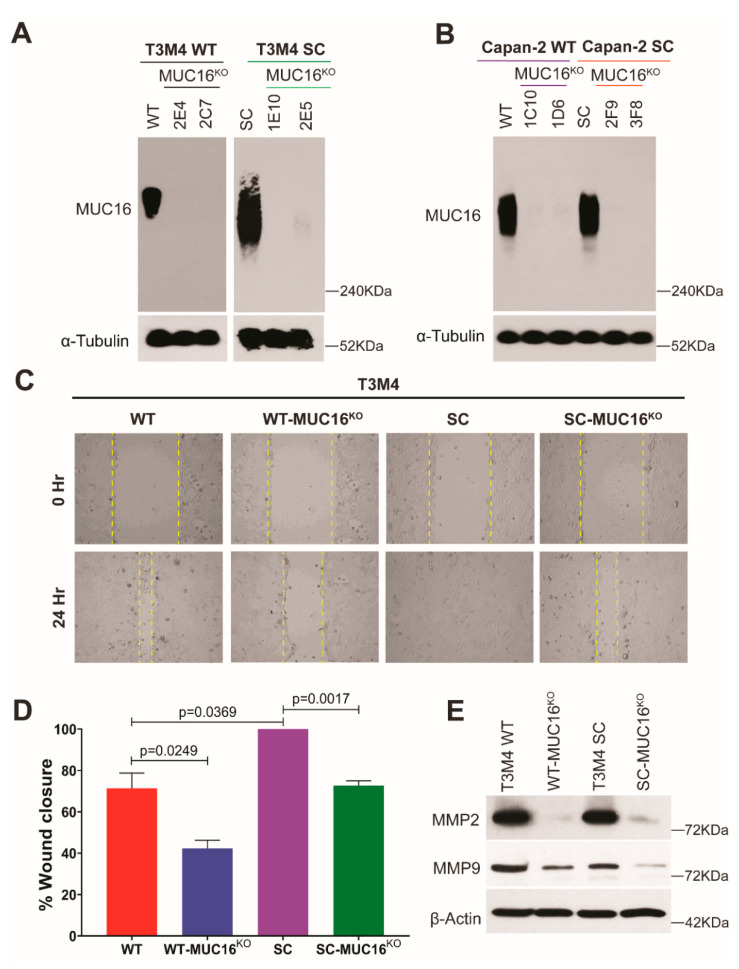
MUC16 knockout causes a decrease in PDAC cell migration. Western blot confirmation of CRISPR-Cas9-mediated genetic deletion of MUC16 in (**A**) T3M4 WT and SC cells; and (**B**) Capan-2 WT and SC cells; loading control: α-tubulin; (**C**) wound healing assay with T3M4 WT, WT-MUC16^KO^, SC, SC-MUC16^KO^ cells; images captured at 0 h and 24 h; (**D**) quantification of the percentage of wound closure, as described previously [14]; *p* < 0.05 is considered statistically significant; (**E**) Western blot analysis of MMP2 and MMP9 in T3M4 WT, WT-MUC16^KO^, SC, SC-MUC16^KO^ cells; loading control: β-actin.

It has been previously demonstrated that truncation of mucin-type O-glycans in clinical specimens of PDAC is the result of reduced expression of Core 1 synthase (C1GalT1) and/or its molecular chaperone, COSMC (Core-1 β3 galactosyltransferase specific molecular chaperone) [26]. Such aberrant expression of truncated O-glycan structures (Tn/STn antigen) on premalignant and malignant cells has been shown to enhance their malignant properties [13,27]. However, the molecular and biological mechanisms by which these truncated O-glycan-containing mucins influence pancreatic tumorigenesis are not well understood. Since it was reported that among various mucins, MUC16 possesses a larger number of glycosites in the large N-terminal proline, threonine, and serine (PTS) region [15], we hypothesized that MUC16 has a vital role in enhancing the oncogenic features of truncated O-glycan-expressing PDAC cells. To decipher the molecular mechanisms in detail, we have first disrupted the *MUC16* gene, and thereby its expression in wildtype (T3M4 WT and Capan-2 WT) and COSMC knockout (COSMC^KO^) truncated O-glycan-expressing (SimpleCells (SC); T3M4 SC and Capan-2 SC) PDAC cells using a CRISPR/Cas9-targeting construct [28]. The knockout of MUC16 in these cells was confirmed by Western blot analysis (Figure 1A,B). We have previously demonstrated that PDAC SC cells increased the cell migratory phenotype [14]. Hence, we used the WT, WT-MUC16^KO^, SC, and SC-MUC16^KO^ cells to investigate the role of truncated O-glycan-containing MUC16 in cell migration. Expectedly, cell migration measured by wound-healing capacity showed that T3M4 WT-MUC16^KO^ cells possessed the lowest migration rate among all the other cells, including their WT counterparts (*p* = 0.0249), highlighting the importance of MUC16 in cell migration. Wound closure was significantly high in T3M4 SC cells (containing truncated O-glycan-bearing MUC16) as compared to WT cells (containing fully branched O-glycan-bearing MUC16) (*p* = 0.0369). Notably, even in the highly aggressive T3M4 SC cells, the deletion of MUC16 resulted in significantly lower cell migration (*p* = 0.0017) (Figure 1C,D). The observed data from the tumor cell migration assay further prompted us to analyze the expression of matrix metalloproteases (MMPs) 2/9, as they are paramount players in the in vivo migration process in cancer [29]. The inherent levels of both MMP2 and MMP9 were highest in the SC cells compared to the WT counterparts. Intriguingly, the loss of MUC16 resulted in a significant decrease in the expression of MMP2 and MMP9 in both WT and SC cells (Figure 1E). Together, these results demonstrate the critical role of truncated O-glycans and truncated O-glycan-containing MUC16 in enhancing cancer cell migration.

### 2.3. Aberrant Glycoforms of MUC16 Enhance PDAC Migratory Function via Activation of Focal Adhesion Kinase (FAK)

**Figure 2:**
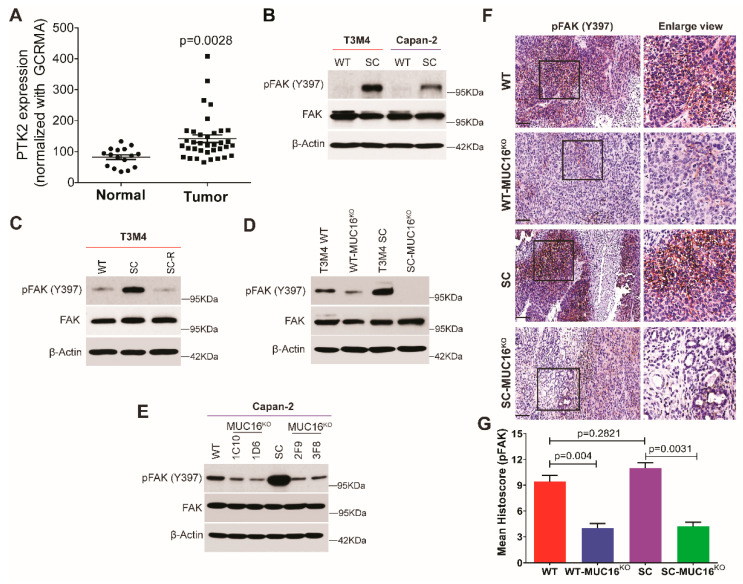
Aberrantly glycosylated MUC16 effectively mediates FAK signaling. (**A**) mRNA expression of the *PTK2* gene (encoding FAK) was determined using microarray data from normal (*n* = 16) and PDAC tumor (*n* = 36) tissues; *p* value < 0.05 is considered statistically significant; Western blot analysis showing (**B**) phosphorylation of FAK at tyrosine 397 (pFAK Y397) and total FAK expression in T3M4 and Capan-2 WT and SC cells; loading control: β-actin; (**C**) re-expression of COSMC in T3M4 SC-R rescues pFAK (Y397) level back to WT parental; loading control: β-actin; (**D**) genetic deletion of MUC16 causes a decrease in pFAK (FAK activation) in T3M4 WT and SC, and (**E**) Capan-2 WT and SC cells; loading control: β-actin; (**F**) immunohistochemistry staining using an antibody for pFAK (Y397) in tumor tissues derived from orthotopic implantation T3M4 WT, WT-MUC16^KO^, SC, SC-MUC16^KO^ cells, as described previously [28]; (**G**) histoscore analysis of pFAK immunohistochemistry showing high pFAK in WT and SC cells and decrease with MUC16^KO^ in both cells. Data representation is as mean ± SEM analyzed using Dunnett’s multiple comparisons test.

We found that aberrant expression of Tn/STn antigens on MUC16 enhanced migratory properties of PDAC cells; however, the underlying mechanism remains elusive. Therefore, we sought to determine whether truncation of O-glycans activates focal adhesion kinase (FAK), a differential regulator of cellular motility and invasiveness. Increased expression of FAK (governed by the *PTK2* gene) is considered to be a prognostic marker in PDAC and is positively correlated with tumor size [30]. We analyzed the expression of FAK by evaluating mRNA expression of *PTK2* in microarray datasets of normal (*n* = 16) and PDAC tumor tissues (*n* = 36) in a cohort published by Pei et al. [31]. As compared to normal tissues, PDAC tumor tissues showed significantly higher levels of FAK expression (*p* = 0.0028) (Figure 2A). This was also validated in our cell line models, wherein increased phosphorylation of FAK at Tyrosine 397 (p-FAK Y397) and its subsequent activation was observed in truncated O-glycan-expressing PDAC cells compared to WT cells (Figure 2B). Further, re-expression of COSMC in truncated O-glycan-expressing PDAC cells (SC-R) (resulting in fully branched O-glycan structures) caused a decrease in expression of p-FAK (Y397) when compared to SC cells (Figure 2C). These results indicate that induced expression of truncated O-glycans enhances migratory properties of PDAC cells via activation of FAK. To determine the role of truncated O-glycan-containing MUC16 in FAK activation, we analyzed the p-FAK levels in WT-MUC16^KO^ and SC-MUC16^KO^ cells. Knock out of MUC16 in T3M4 WT and SC cells showed a significant decrease in FAK phosphorylation (Y397) (Figure 2D). A similar reduction in FAK phosphorylation was also observed in Capan-2 cells upon loss of MUC16 (Figure 2E), demonstrating the importance of truncated O-glycan-expressing MUC16 in this model. Further, immunohistochemistry staining of p-FAK in T3M4 WT, WT-MUC16^KO^, SC, and SC-MUC16^KO^ cells implanted tumor tissues (as per the orthotopic tumor model described in [28]) revealed a significant downregulation in the expression of p-FAK in T3M4 WT- MUC16^KO^ cells (*p* = 0.004) as well as T3M4 SC-MUC16^KO^ cells (*p* = 0.0031) implanted groups as compared to WT and SC cells implanted tumors, respectively (Figure 2F,G). Altogether, these results support the hypothesis that aberrant expression of truncated O-glycans on MUC16 in PDAC cells enhances migratory properties of these cells via activation of FAK.

### 2.4. Aberrantly Glycosylated MUC16 Strongly Interacts with α4β1 Integrins

**Figure 3:**
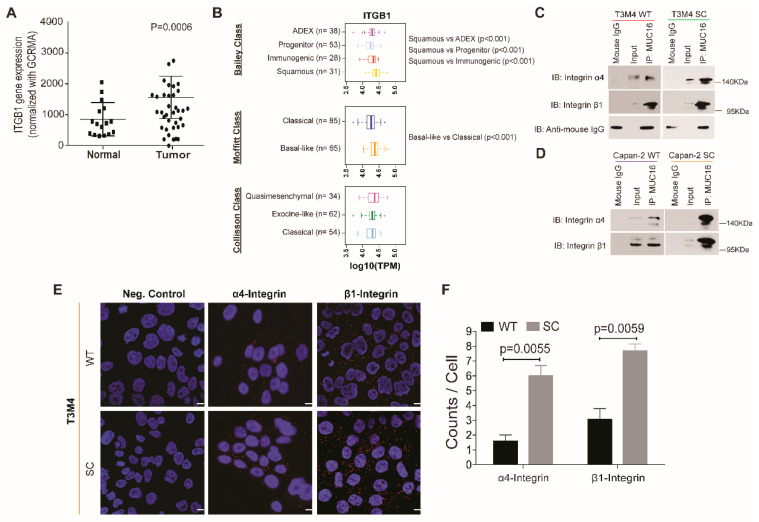
Truncated O-glycan-containing MUC16 interacts with α4β1 integrins. (**A**) mRNA expression of the *ITGB1* gene (encoding Integrin β1) was determined using microarray data from normal (*n* = 16) and PDAC tumor (*n* = 36) tissues; *p* value < 0.05 is considered statistically significant; (**B**) box plots showing *ITGB1* expression level stratified by mRNA expression data from Bailey class, Moffitt class, and Collisson class of PDAC; box plots were generated by comparing the expression of the ITGB1 gene among the subtypes indicated by using the Mann–Whitney rank-sum test; *p* value < 0.01 is considered statistically significant; immunoprecipitation analysis in (**C**) T3M4 WT and SC cells and (**D**) Capan-2 WT and SC cells show an association of MUC16 with integrin α4 and integrin β1 (IP: immunoprecipitation, IB: immunoblot); (**E**) proximity ligation assay (PLA) using antibodies for MUC16, integrin α4, and integrin β1 in T3M4 WT and SC cells; scale bar is equivalent to 10 µm. (**F**) quantification of the interaction as counts per cell using the blob finder 2 software; negative control: incubation of cells with antibodies against a single antigen; data presented as mean ± SEM and analyzed using the unpaired t test (*n* = 3); *p* < 0.05 is considered statistically significant.

Several studies have established that activation of FAK is regulated by the receptor–ligand interactions between integrin complexes and extracellular matrix components on the cell surface [32,33]. Using the publicly available TCGA-PAAD dataset, the correlation between mRNA expression of *MUC16* and β1 integrin (encoded by the *ITGB1* gene) resulted in a positive Pearson correlation coefficient of 0.26 and Spearman correlation of 0.47 (Supplementary Figure S1D) [23]. The CPTAC proteomic dataset also revealed a significant upregulation in the expression of the β1 integrin in high-grade tumors, seemingly representing all stages of PDAC (Supplementary Figure S1E,F) [21]. Y397 phosphorylation of FAK, as observed in our study, has also been shown to be a downstream effect of integrin signaling [34]. In the microarray dataset [31] used to study FAK expression, the mRNA expression of the β1 integrin was also found to be significantly higher (*p* = 0.0006) in PDAC tumor tissues (*n* = 36) as compared to normal tissues (*n* = 16) (Figure 3A). We then analyzed the expression of *ITGB1* in tumor subtypes stratified from the TCGA-PAAD cohort [23]. Elevated *ITGB1* mRNA expression was observed in: (a.) Bailey’s classification-based Squamous subtype when compared to the ADEX (Aberrantly differentiated endocrine-exocrine), progenitor, and immunogenic subtypes (*p* < 0.001); (b.) Moffitt-classification-based basal-like subtype as compared to classical subtype (*p* < 0.001); and (c.) Collisson-classification-based quasi-mesenchymal subtype of PDAC as compared to the exocrine-like and classical subtypes (Figure 3B). This trend of *ITGB1* gene expression mimics that of *MUC16* mRNA expression in the three classifications of PDAC, as reported in a previous study [28]. It is important to note that the subtypes highlighted for high *ITGB1* (and *MUC16* as described in [28]) gene expression, namely the squamous (Bailey’s class), basal-like (Moffitt class), and quasimesenchymal (Collisson class) subtypes, are known to be associated with worsened survival and poor overall prognosis in PDAC patients [35]. We thus hypothesized that interactions between aberrant glycoforms of MUC16 and integrin complexes are responsible for activating FAK in truncated O-glycan-expressing PDAC cells. Co-immunoprecipitation studies of several integrins (α2, α4, α5, β1, β3, β4, and β5) with MUC16 in T3M4 WT and SC cells revealed that only α4 and β1 integrins demonstrate strong interactions with aberrant glycoforms of MUC16 in SC cells (Figure 3C). Studies with Capan-2 cells also showed strong interactions between MUC16 and α4β1 integrins in SC cells compared to WT (Figure 3D). To validate such an interaction, the proximity ligation assay (PLA) was employed, wherein the WT and SC cells were probed for MUC16, α4, and β1 integrins using target-specific antibodies. The PLA revealed a significantly higher interaction between MUC16:α4 integrin and MUC16:β1 integrin complexes in T3M4 SC cells than in WT cells (Figure 3E). Quantitative analysis (signals/dots per cell analyzed through blob finder software) of these interactions between MUC16 and α4 integrin (*p* = 0.0055) and β1 integrin (*p* = 0.0059) confirmed their higher proximity in SC cells as compared to the parental WT cells (Figure 3F). This data demonstrates that truncated O-glycan-containing MUC16 interacts with the α4 and β1 integrins and mediates their functional activation in PDAC.

### 2.5. Integrin α4β1–ILK–FAK Axis Is Upregulated in PDAC Cells with Truncated O-Glycan Containing MUC16

**Figure 4:**
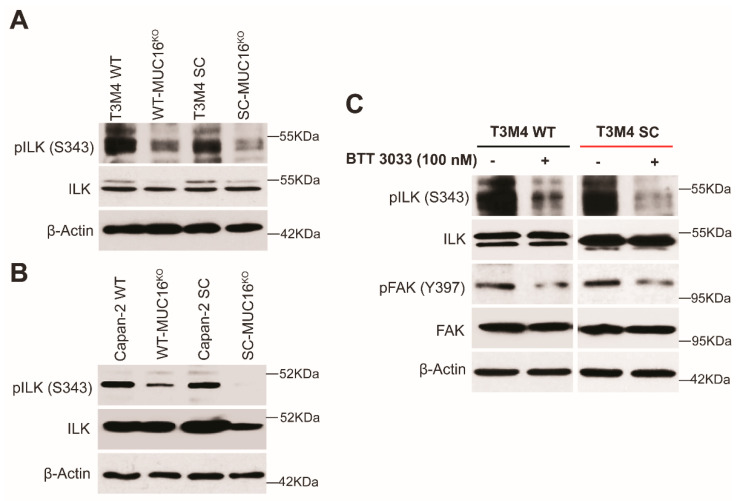
Truncated O-glycan-bearing MUC16 potentiates integrin signaling. Western blot analysis showing (**A**) phosphorylation of ILK at Serine 343 (pILK S343) and total ILK in T3M4 WT, WT-MUC16^KO^, SC, SC-MUC16^KO^ cells; loading control: β-actin and in (**B**) Capan-2 WT, WT-MUC16^KO^, SC, SC-MUC16^KO^ cells; loading control: β-actin; (**C**) pILK (S343) and pFAK (Y397) in T3M4 WT and SC cells treated with the integrin inhibitor, BTT 3033 (100 nM), for 24 h (control: untreated); loading control: β-actin.

To further validate the role of FAK signaling and the possible mediators of this pathway in our model, we evaluated the expression of integrin-linked kinase (ILK)—a crucial positive modulator in the integrin-FAK axis [36]. We found that ILK was highly activated via increased phosphorylation (S343) in T3M4 and Capan-2 SC cells as compared to WT, while the loss of MUC16 caused a decrease in such phosphorylation (Figure 4A,B). To further confirm that integrins activate FAK signaling through ILK, we exposed T3M4 (WT and SC) cells to a selective small-molecule integrin inhibitor, BTT 3033, at a concentration of 100 nM for 24 h [37]. Treatment of cells with BTT 3033 resulted in the inhibition of phosphorylation of ILK at S343 in both T3M4 WT as well as SC cells (Figure 4C). Interestingly, a significant downregulation was observed in the phosphorylation of FAK in BTT 3033 treated T3M4 WT and SC cells (Figure 4C). This finding supports the hypothesis that integrins regulate the activation of FAK signaling through ILK. Overall, these results suggest that the interaction between MUC16 and α4β1 integrins increases the migratory ability of PDAC cells, and these interactions are positively regulated by aberrantly glycosylated structures frequently observed in this cancer.

### 2.6. Anti-MUC16 Antibody (AR9.6) Treatment Reduces Downstream Integrin-FAK Signaling

**Figure 5:**
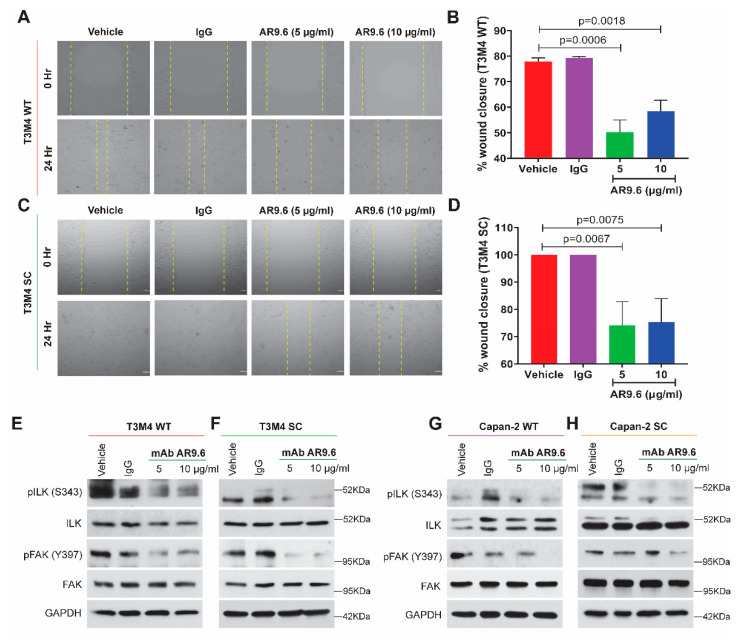
Treatment with anti-MUC16 AR9.6 antibody alleviates PDAC cell migration via the ILK-FAK axis. Cells were treated with anti-MUC16 AR9.6 antibody (5 µg/mL and 10 µg/mL); controls: untreated (complete media) and isotype-control (5 µg/mL murine IgG) for all of the following: wound healing assay with (**A**) T3M4 WT cells; (**B**) quantification of percent wound closure in T3M4 WT cells; (**C**) T3M4 SC cells; (**D**) quantification of percent wound closure in T3M4 SC cells; *p* < 0.05 is considered statistically significant. Western blot analysis for pILK (S343), total ILK, pFAK (Y397), total FAK in (**E**) T3M4 WT; (**F**) T3M4 SC; (**G**) Capan-2 WT; (**H**) Capan-2 SC cells; loading control: GAPDH.

We have previously demonstrated that the MUC16-targeting AR9.6 antibody robustly inhibits MUC16 dependent signaling and reduces the tumor burden in orthotopic mouse models of PDAC [28]. To evaluate if such antibody-mediated MUC16 inhibition affects the cell migration and, more specifically, the integrin-FAK axis, T3M4, and Capan-2 (WT and SC) cells were treated with the AR9.6 antibody at concentrations of 5 µg/mL and 10 µg/mL for 24 h, as previously reported [28]. An isotype control murine IgG was used at 5 µg/mL under the same conditions. The wound-healing assay revealed a decrease in wound closure and cell migration in the AR9.6 antibody-treated groups compared to the untreated and isotype IgG treated controls in WT (Figure 5A) and SC cells (Figure 5C). Further, quantification of wound closure revealed a significant decrease in migration in T3M4 WT cells treated with 5 µg/mL AR9.6 (*p* = 0.006) and 10 µg/mL AR9.6 (*p* = 0.0018) (Figure 5B) and SC cells treated 5 µg/mL AR9.6 (*p* = 0.0067) and 10 µg/mL AR9.6 (*p* = 0.0075) (Figure 5D). The western blotting analysis confirmed the downregulation of Y397 FAK phosphorylation and S343 ILK phosphorylation upon AR9.6 treatment in T3M4 WT and SC cells (Figure 5E,F), as well as in Capan-2 WT and SC cells (Figure 5G,H). Thus, these results strongly demonstrate the importance of MUC16 and the truncated O-glycan structure on this mucin in mediating integrin-FAK-mediated cell migration in PDAC.

## 3. Discussion

The highest occurrence of aberrant glycosylation patterns has been observed in PDAC amongst the various solid tumors assessed [12]. This study was undertaken to understand the molecular mechanisms by which such aberrant glycosylation motifs in glycoproteins induce oncogenic features in PDAC cells. The role of MUC16 in promoting tumorigenicity has been widely demonstrated in ovarian and breast cancers, as has been recently extended to PDAC [38,39,40,41]. Herein, we employ CRISPR-Cas9 to genetically delete MUC16 in truncated O-glycan-expressing PDAC cells and demonstrate a dampening of tumorigenicity in these cells. A hallmark of PDAC pathogenesis, like most solid tumors, is the high potential for metastatic spread facilitated through complex coordination amongst diverse cellular processes, including cell migration and cell–cell and cell–matrix adhesion-related pathways in the tumor niche [42]. Decreased tumor cell migration and invasion upon deletion of MUC16 in T3M4 WT and SC cells highlight the tumorigenic potential of MUC16 [28]. Moreover, the least migration and invasion rate in T3M4 SC-MUC16^KO^ cells provides further impetus to the theory that aberrant glycosylation is an important player that enhances the ability of MUC16 to affect the tumorigenicity of PDAC cells [14,27].

To investigate the molecular events induced by aberrant glycosylation and thereby truncated O-glycan containing MUC16, we hypothesized that altered glycosylation of mucins influences their capacity to interact with integrins and cause the subsequent modulation of cell migration events that play a pivotal role in carcinogenesis. This study demonstrates that oncogenic interactions exist between aberrant glycoforms of MUC16 as seen in the SC cells and integrin α4β1 complexes, thereby unraveling a key and novel mechanism underlying the activation of FAK signaling to facilitate the increased migratory/invasive properties of PDAC cells.

**Figure 6:**
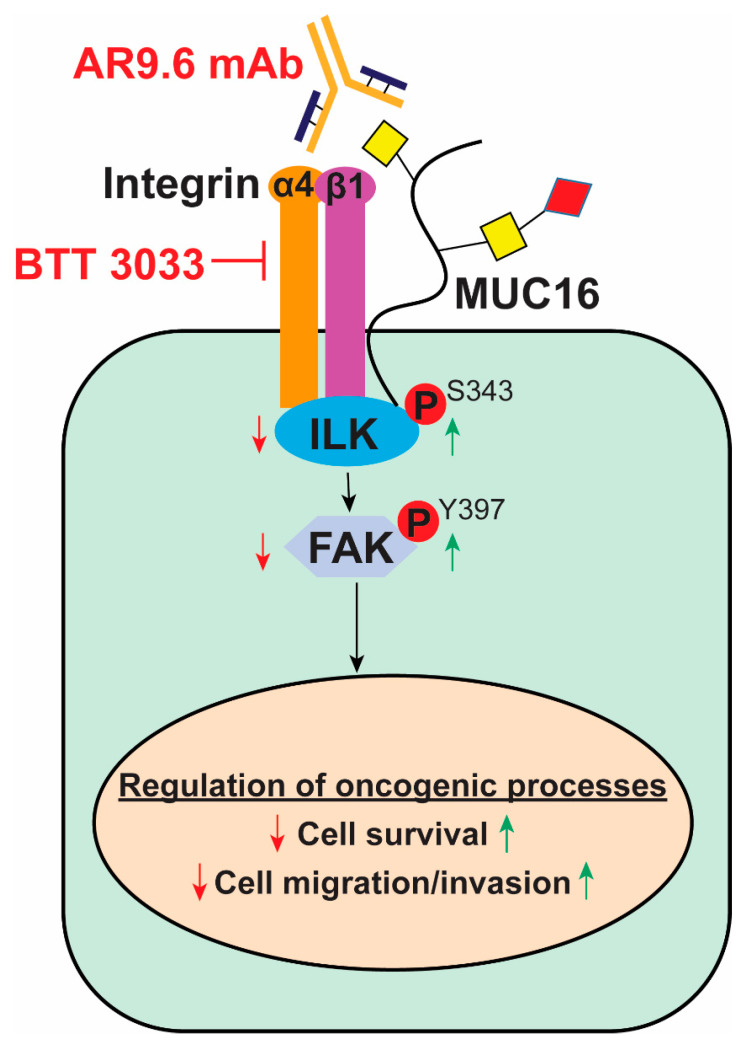
The proposed model for truncated O-glycan-containing MUC16 facilitates α4β1 integrin signaling. Interaction between Tn/STn-MUC16 and the α4β1 integrin complex facilitates activation of the integrin-linked kinase (ILK) and focal adhesion kinase (FAK) axis to increase migratory behavior in PDAC (indicated by green arrows). Blocking the integrin signaling using the BTT 3033 inhibitor or MUC16 signaling using the monoclonal antibody, AR9.6, dampens the ILK-FAK cascade and decreases cell migration (indicated by red arrows).

Activation of intracellular kinases ILK/FAK has long been associated with the migration of cancer cells by promoting dynamic regulation of focal adhesions and peripheral actin structures [43]. Interactions between the extracellular matrix and integrin complexes and activation of ILK are known prerequisites for activation of FAK in multiple tumor models [44]. We found that aberrant expression of truncated O-glycans in SC cells tremendously upregulates the phosphorylation of ILK/FAK and exacerbates cancer progression. In this study, we provide the first experimental evidence that MUC16 can interact with α4/β1 integrins, and these interactions are significantly higher with aberrant glycoforms of MUC16. Our study shows that PDAC cell migration can partially be explained by increased interactions between aberrant glycoforms of MUC16 and the non-RGD-binding integrin α4/β1 complexes in PDAC tumors expressing truncated O-glycans. Using the AR9.6 monoclonal antibody to block MUC16 on these PDAC cells, the MUC16/integrin-mediated ILK and FAK activation was able to be suppressed, providing a therapeutic opportunity encompassing anti-MUC16 strategies (Figure 6).

## 4. Materials and Methods

### 4.1. Cell Lines and Culture

Isogenic PDAC cells (T3M4 and Capan-2) wild type (WT), COSMC Knockout SimpleCells (SC), and COSMC re-expression (SCR) were produced and maintained, as described previously [14].

### 4.2. Genetic Deletion of MUC16 in PDAC Cells

The N-terminal region of the *MUC16* gene was genetically deleted in WT and SC PDAC cells (T3M4 and Capan-2) by using pre-made CRISPR/Cas9 KO plasmid kits, as described previously [28]. These MUC16 knockout cells (T3M4 WT-MUC16^KO^, T3M4 SC-MUC16^KO^, Capan-2 WT-MUC16^KO^, and Capan-2 SC-MUC16^KO^) were utilized for further experiments.

### 4.3. Wound-Healing Assay

T3M4 WT, T3M4 WT-MUC16^KO^, T3M4 SC, and T3M4 SC-MUC16^KO^ cells were seeded onto six-well plates. After cells attained 80–90% confluency in a monolayer, the scratch/wound was created using a 200 µl pipet tip. Plates were washed with 1X PBS and replenished with a complete DMEM medium, following which the images for the 0 h time point were captured using bright-field microscopy. For antibody treatment conditions, control murine IgG (5 µg/mL) (Jackson Immunoresearch Laboratories, Inc., West Grove, PN, USA) and murine AR9.6 (5 µg/mL and 10 µg/mL) (Quest PharmaTech Inc., Edmonton, AB, Canada) were added to complete the media after the 0 h timepoint. The cells were incubated at 37 °C, 5% CO_2_ for 24 h, and images were recaptured at the same position as 0 h. The distance between the scratches was measured in µm and wound healing was calculated, as described previously [14].

### 4.4. Western Blotting

T3M4 and Capan-2 (WT, WT-MUC16^KO^, SC, SC-MUC16^KO^) whole cell lysates [RIPA lysis buffer (Thermo Scientific, Rockford, IL USA) with protease and proteinase inhibitor, 30 µg/lane] were resolved on 1.5% SDS-Agarose for the detection of MUC16, as described in [28], and 4–20% SDS-PAGE for all other markers, transferred to PVDF membrane (Merck Millipore, Burlington, MA, USA). For the integrin inhibitor treatment conditions, T3M4 and Capan-2 (WT and SC) cells were treated with BTT 3033 (Tocris Biosciences, Minneapolis, MN, USA) at 100 nM for 24 h. For antibody treatment conditions, control murine IgG (5 µg/mL) (Jackson Immunoresearch Laboratories, Inc., PN, USA) and murine AR9.6 (5 µg/mL and 10 µg/mL) (Quest PharmaTech Inc., AB, Canada) were added to complete media and incubated as above for 24 h. Cells were lysed after the treatment period, and protein lysates were resolved on SDS-PAGE, as described above. The membranes were probed with the anti-MUC16 AR9.6 (Quest PharmaTech Inc., AB, Canada), anti-α-tubulin (Developmental studies hybridoma bank, IA, USA), anti-phospho-FAK (Y397), anti-FAK, anti-MMP-2, anti-MMP-9, anti-GAPDH, anti-β-actin (Cell Signaling Technology, Danvers, MA, USA), anti-phospho ILK (S343), and anti-ILK (Thermo Fisher Scientific, Rockford, IL, USA) antibodies. The membranes were then probed with the respective HRP conjugated goat anti-mouse IgG and goat anti-rabbit IgG (Cell Signaling Technology, Danvers, MA, USA). The signal was detected using the Clarity^TM^ Western ECL Chemiluminescent kit (Bio-Rad, Hercules, CA, USA).

### 4.5. Stratification of ITGB1 Expression in PDAC Subtypes

Clustering of TCGA PAAD dataset samples (*n* = 150) into subtypes was done, as described previously [35]. *ITGB1* expression and stratification based on these PDAC subtypes was done and analyzed using the Mann–Whitney rank-sum test [28].

### 4.6. Immunoprecipitation Analysis

T3M4 and Capan-2 (WT and SC) cells were lysed in non-denaturing RIPA buffer (Thermo scientific, Lenexa, KS, USA) containing protease inhibitor (Roche Diagnostics GmbH, Mannheim, Germany). A total of 500 µg of proteins were incubated with either mouse anti-MUC16 (5 µg/mL) or mouse IgG (5 µg/mL; Jackson ImmunoResearch Laboratories, Inc., PN, USA) overnight at 4 °C. Lysates were then incubated with protein G Sepharose beads (GenScript, Piscataway, NJ, USA) for 2 h at room temperature. Beads were collected, washed three times with RIPA buffer without a protease inhibitor, and boiled in SDS loading buffer (1X). Samples were subjected to 4–20% gradient (Bio-Rad, CA, USA) SDS-PAGE gel electrophoresis and transferred to the PVDF membrane. The membranes were blocked with 5% skimmed milk and incubated with primary antibodies rabbit anti-integrin α4 (Cell Signaling Technology, Danvers, MA, USA, 1:1000) and rabbit anti- integrin β1 (Cell Signaling technology, Danvers, MA, USA, 1:1000) overnight. After incubating with the respective secondary antibodies, the protein–antibody complex was detected using enhanced chemiluminescence (Bio-Rad, CA, USA).

### 4.7. Proximity Ligation Assay

The protein–protein interaction experiments (growth factor receptors interactions with mucins and integrin complexes) were performed by using Duo Link II (Olink Bioscience, Watertown, MA, USA) Proximity Ligation Assay kit according to the manufacturer’s recommendation, as described previously [28]. Briefly, T3M4 WT and SC cells were grown on coverslips and then incubated with primary antibodies specific to MUC16 (AR9.6, Quest PharmaTech, Inc., AB, Canada), α4-integrin (Cell Signaling Technology, Danvers, MA, USA), and β1-integrin (EMD Millipore, Billerica, MA, USA), and then the primary antibodies were incubated with PLA probes anti-rabbit PLUS, anti-mouse MINUS. Experiments omitting the primary antibody used for single recognition PLA or either one of the primary antibodies used in double recognition PLA served as a negative control. The fluorescence images were captured under a Zeiss LSM 510 laser scanning confocal microscope (Carl Zeiss, Inc., Thornwood, NY, USA) at Confocal Laser Scanning Fluorescence Microscopy Core Facility, UNMC. The signal/dots per cell were quantified by using blob finder software.

### 4.8. Immunohistochemistry

T3M4 WT, WT-MUC16^KO^, SC, and SC-MUC16^KO^ cells orthotopically implanted tumor tissues from a previous study [28] were formalin-fixed and paraffin-embedded to prepare tissue slides. Slides were de-paraffinized using xylene, sequentially re-hydrated with alcohol (100%, 90%, 80%, 70%) and water, followed by hydrogen peroxide for quenching, and antigen retrieval in citrate buffer (pH = 6.0) for 10 min. They were blocked with Universal Blocker (Thermo Fisher Scientific, Waltham, MA, USA) and incubated with primary antibodies, anti-phospho-FAK (Thermo Fisher Scientific, Waltham, MA, USA), at 4 °C overnight. After washing with 1X TBS, slides were incubated with the anti-rabbit HRP-conjugated secondary antibody (Agilent, CA, USA) for 1 h at room temperature. Post-washing, tissues were exposed to 3,3′-diaminobenzidine tetrahydrochloride (DAB, Vector Laboratories, Burlingame, CA, USA) substrate and counterstained with hematoxylin. It was followed by sequential dehydration with alcohol and cleared with xylene. Slides were mounted in mounting media with a coverslip. Histoscoring was done by a board-certified pathologist. Histoscore quantification was completed, as described previously [28].

### 4.9. Statistical Analysis

In vitro tumor cell migration was analyzed between T3M4 WT, WT-MUC16^KO^, T3M4 SC, and SC-MUC16^KO^ cells by unpaired Student’s *t* test. The statistical significance in the protein expression between groups was analyzed by two-way ANOVA. *p* < 0.05 was considered statistically significant.

## 5. Conclusions

In summary, our findings demonstrate that novel oncogenic interactions between aberrant glycoforms of MUC16 and integrin (α4 and β1) complexes enhance the migratory and pro-tumorigenic properties of PDAC cells via constitutive activation of FAK and its mediators. These findings can be used as a model to study and therapeutically target the truncated O-glycan-containing MUC16/integrin/ILK/FAK-signaling cascade to develop treatments against metastatic pancreatic cancer (Figure 6).

## Data Availability

The data presented in this study are available on request from the corresponding authors.

## Supplementary Materials

The following supporting information can be downloaded at: https://www.mdpi.com/article/10.3390/ijms23105459/s1.
