# Supplementary material for: Truncated O-Glycan-Bearing MUC16 Enhances Pancreatic Cancer Cells Aggressiveness via α4β1 Integrin Complexes and FAK Signaling
Source: Int J Mol Sci. 2022 May 13;23(10):5459. doi: 10.3390/ijms23105459 (PMC9141077; doi:10.3390/ijms23105459)
Supplement: Supplementary file 1 [file ijms-23-05459-s001.zip › Supplementary Figure 1.pdf]

Supplementary Figures

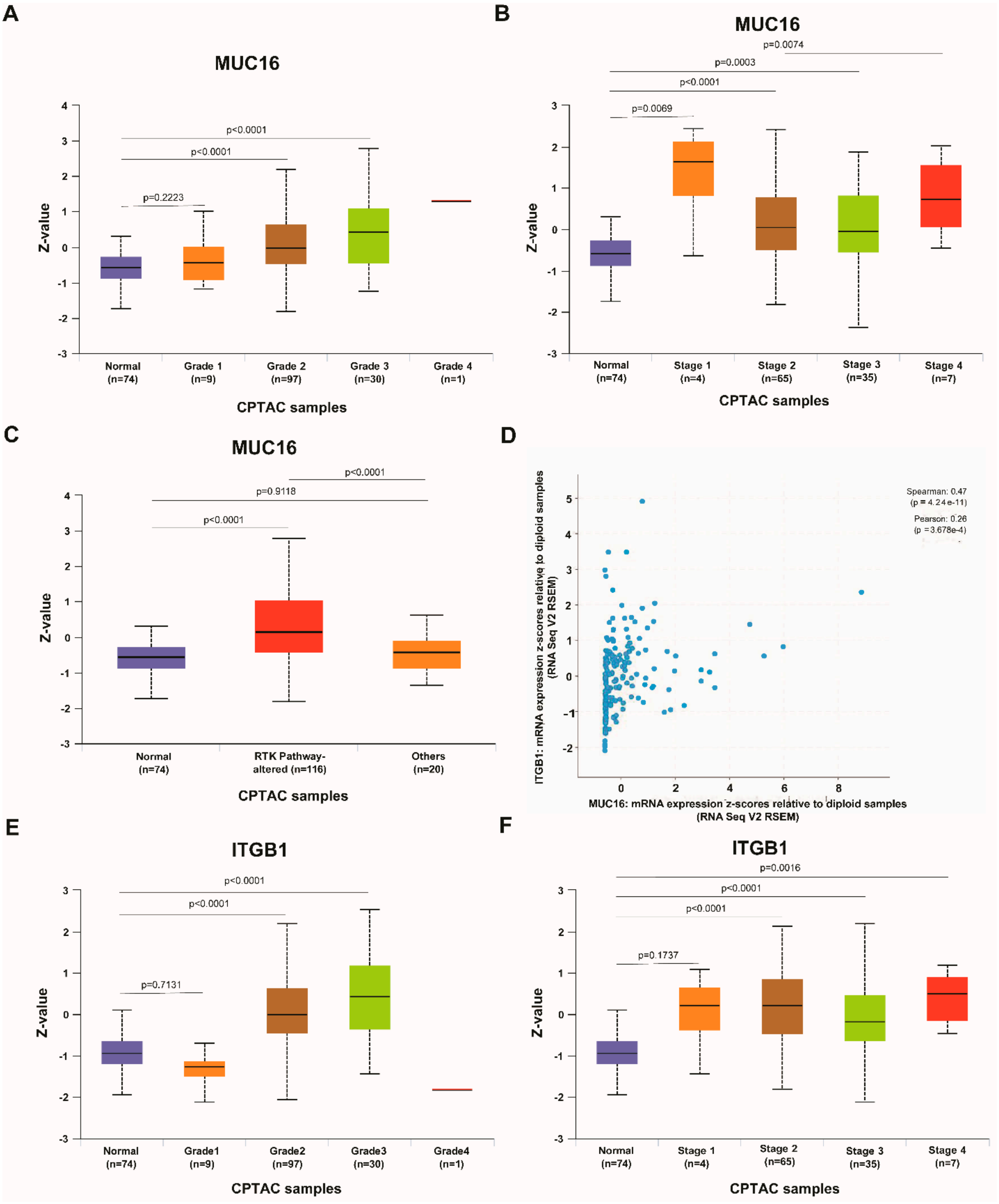

Supplementary Figure 1: In silico analysis of MUC16 and ITGB1 expression in PDAC (A) MUC16 protein expression stratification by tumor grade, (B) Tumor stage, and (C) Receptor

tyrosine kinase (RTK) pathway protein alteration, using the cohorts from the Clinical Proteomic Tumor Analysis Consortium (CPTAC) dataset, (D) Comparison of mRNA expression profiles (n=73 samples, 7% of total 177 queried cases from the TCGA-PAAD cohort) of *ITGB1* against *MUC16* in unaltered (diploid) samples without copy number variations. Highlighted Pearson Correlation Coefficient (0.26) represents positive linearity, and Spearman Correlation Coefficient (0.47) represents the positive monotonic relationship between the expression of the two genes, (E) ITGB1 protein expression stratification by tumor grade, (F) Tumor stage. n represents the number of samples. Z-value is a measure of standard deviations from the median in all samples of PDAC. Log2 spectral count ratios obtained from CPTAC have been normalized against each sample first and then across all samples; $p < 0.05$ is considered statistically significant.
